# CA9-Related Acidic Microenvironment Mediates CD8+ T Cell Related Immunosuppression in Pancreatic Cancer

**DOI:** 10.3389/fonc.2021.832315

**Published:** 2022-01-27

**Authors:** Lingdi Yin, Yichao Lu, Cheng Cao, Zipeng Lu, Jishu Wei, Xiaole Zhu, Jianmin Chen, Feng Guo, Min Tu, Chunhua Xi, Kai Zhang, Junli Wu, Wentao Gao, Kuirong Jiang, Yi Miao, Qiang Li, Yunpeng Peng

**Affiliations:** ^1^ Pancreas Center, The First Affiliated Hospital of Nanjing Medical University, Nanjing, China; ^2^ Pancreas Institute of Nanjing Medical University, Nanjing, China

**Keywords:** pancreatic cancer, tumor microenvironment, CA9, CD8+ T cells, immunotherapy

## Abstract

**Purpose:**

This study aims to integrate pancreatic cancer TCGA, GEO, and single-cell RNA-sequencing (scRNA-seq) datasets, and explore the potential prognostic markers and underlying mechanisms of the immune microenvironment of pancreatic cancer through bioinformatics methods, *in vitro* and *in vivo* assays.

**Methods:**

Expression data and clinicopathological data of pancreatic cancer TCGA, GEO (GSE131050), single cell sequencing (PAAD_CRA001160) dataset were downloaded. We used R/Bioconductor edgeR for differential expression analysis. ClusterProfiler was utilized to perform GO enrichment analysis on differentially expressed genes. The online software CIBERSORT was used to reanalyze the mRNA expression data of pancreatic cancer. CellRanger, RunPCA, FindNeighbors, FindClusters, RunTSNE and RunUMAP were used to perform preprocessing, cell clustering and expression profile analysis on single-cell sequencing data sets. We analyzed intracellular pH with or without CA9 inhibitor SLC-0111. Indirect co-culture model of human pancreatic cancer cell lines and healthy individual-derived PBMCs were used to determine the effect of CA9-related Acidic Microenvironment on CD8+ T cells.

**Results:**

The CIBERSORT analysis of TCGA pancreatic cancer transcriptome sequencing data showed that among the 22 immune microenvironment components, CD8+ T cell infiltration was significantly correlated with the prognosis of pancreatic cancer patients. The differential expression analysis of the TCGA data grouped by the level of CD8+ T cell infiltration indicates that the expression of carbonic anhydrase 9 (CA9) is the most significant, and the survival analysis suggests that CA9 is associated with the overall survival of pancreatic cancer. TCGA data and GEO data set GSE131050 expression correlation analysis suggests that CA9 and CD8 expression are closely related. Pancreatic cancer single-cell sequencing data set PAAD_CRA001160 analysis results show that CA9 is mainly expressed in pancreatic cancer cell clusters, and the expression of the cancer cell subgroup CA9 in the single-cell data set is correlated with CD8+ T cell infiltration.

**Conclusion:**

Pancreatic cancer cells may inhibit the infiltration of CD8+ T cells through CA9. Further exploration of its related mechanisms can be used to explore the immune escape pathway of pancreatic cancer and provides new perspectives immune targeted therapy.

## Introduction

Pancreatic cancer is one of the solid tumors with the worst prognosis, and the incidence in China is increasing year by year (1). The onset of pancreatic cancer is insidious, and more than 80% of patients lose the opportunity to undergo radical resection due to local progression and/or distant metastasis at the time of diagnosis (2), while patients undergoing radical resection are usually observed with local or distant recurrence within two years after surgery (3). In addition to surgical resection, chemotherapy is also greatly restricted in the treatment of pancreatic cancer due to the extremely high drug resistance rate. In recent years, immunotherapy has become the most promising treatment for pancreatic cancer other than surgery and chemotherapy. However, due to the strong heterogeneity of the immune microenvironment of pancreatic cancer, and the formation of the immunosuppressive microenvironment is not clear, the current immunotherapy has limited efficacy in pancreatic cancer (4).

Our study found that the lack of CD8+ T cells in the immune microenvironment of pancreatic cancer is the most significant risk factor for its poor prognosis, and cancer cells may inhibit CD8+ T cell infiltration through CA9 related mechanism, which provides new ideas for exploring pancreatic cancer immune escape pathways and immune targeted therapy.

## Materials And Methods

### Data Source

Pancreatic cancer mRNA expression data and single-cell RNA-sequencing of TCGA, GEO, database gene were downloaded from TCGA (https://xenabrowser.net/datapages/), CBioPortal (https://www.cbioportal.org/datasets) and Gene Expression Omnibus (GEO) (http://www.ncbi.nlm.nih.gov/geo/). In TCGA database, patients with OS shorter than 1 month or with rare pathological type as colloid carcinoma and undifferentiated carcinoma. 154 patients of TCGA cohort were included for further analysis. GEO dataset GSE131050 (GPL11154 Illumina HiSeq 2000 (Homo sapiens) with 47 PDAC samples were selected to validate the results of TCGA cohort (5). The single-cell RNA-seq (6) dataset was downloaded from Genome Sequence Archive (accession number: CRA001160) at https://bigd.big.ac.cn/bioproject/browse/PRJCA001063.

### Bioinformatical Methodology

Gene differential expression analysis were performed using R/Bioconductor edgeR. Firstly, low-expressed genes and miRNAs were filtered with count per million (cpm)≤1 in more than 10% of the samples. The threshold of differential expression is FDR<0.05, fold-change>1.5. Pathway enrichment was conducted using clusterProfiler including biological process (BP), molecular function (MF) and cellular component (CC), and KEGG signal pathway enrichment analysis. The enrichment significance threshold is FDR<0.05. For the significantly enriched KEGG pathway, pathview was used to display the pathway. Online software CIBERSORT (https://cibersort.stanford.edu/index.php) was used to reanalyze the mRNA expression data of pancreatic cancer for immune microenvironment components. CellRanger, RunPCA, FindNeighbors, FindClusters, RunTSNE, and RunUMAP were used to perform preprocessing, cell clustering and expression profile analysis on the single-cell sequencing dataset PAAD_CRA001160.

### Cell Culture

Human PDAC cell lines (PANC-1, CFPAC-1) were obtained from the American Type Culture Collection (ATCC; Rockville, MD, USA). and were cultured using standard techniques. Pancreatic cancer cell lines were cultured in DMEM supplemented with 10% FBS, penicillin (100 U/mL) and streptomycin (100 μg/mL). Cells were maintained at 37°C in a humidified incubator with 5% CO2.

Peripheral blood mononuclear cells (PBMC) were isolated as previously described (7). Pancreatic cancer cell lines and the isolated PBMCs were cultured in DMEM, supplemented with 10% FBS, 100U/ml penicillin, and 100 μg/ml streptomycin at 37°C in a 5% CO2, 95% air environment in humidified incubators with or without CA9 inhibitor SLC-0111.

### qPCR

Total RNA was extracted from PDAC cell lines (CFPAC-1, PANC-1) with or without co-incubation with SLC-0111 using Trizol reagent and reverse-transcribed into cDNA with PrimeScript RT Master Mix. The process of qRT-PCR amplification was performed using the Step One Plus Real-Time PCR System (Applied Biosystems, Carlsbad, CA, USA) with FastStart Universal SYBR Green Master. All the experiments mentioned here were performed according to relevant manufacturer’s instructions. The formula 2-ΔΔCt (Ct means the cycle threshold) was used to normalize the relative expression of mRNA in certain cells.

### Western Blotting

Total protein was extracted from PDAC cell lines (CFPAC-1, PANC-1) with or without co-incubation with SLC-0111 by using a lysis buffer containing PMSF, protease inhibitors, and phosphatase inhibitors (1 mL lysis buffer with 5μl 100mM PMSF, 1μL protease inhibitors, and 5μL phosphatase inhibitors). Protein lysates from cells were subjected to 5×SDS-PAGE. Western blot analysis was performed according to standard methods.

### *In Vivo* Model

Mouse pancreatic cancer cells PANC02 (dissolved in PBS, 2×10^7^/100μL, 100 μL/per nude mice) were injected subcutaneously in the right flank of C57BL/6 mice. After tumor volume reached 150–200 mm3, 12 tumor-bearing mice were randomly divided into two groups (control group, CA9 group). SLC-0111 (50 mg/kg) and vehicle were administered daily then. Changes in mouse body weight were monitored throughout the study, tumor growth was observed every 2 days and was calculated by the formula: tumor volume=length×width×height/2. Mice were sacrificed at day 22, tumors were excised, weighed, and volume was measured for between group comparisons. Relative inhibition ratio was calculated by the formula: [(volume1-volume2)/volume1] ×100%.

### Immunofluorescence

We measured the expression level of CA9 using multi-color immunofluorescence in the mice tumor slides. CA9 (FITC) and CD8A (Cy3) were simultaneously stained in serial sections. In short, antigen retrieval was performed with citrate buffer pH 8.0 for 25 min at 97° C in a pressure-boiling container. Blocking was subsequently conducted using 0.3% bovine serum albumin in 0.05% Tween solution for 30 min, followed by incubation with primary antibodies at 4° C overnight. At last, fluorescein-labeled secondary antibodies were added for signaling detection at room temperature. Nuclei were detected

### Tissue Microarray (TMA) Construction

A total of 79 samples of PC patients were collected in The First Affiliated Hospital of Nanjing Medical University. All PC patients were regularly followed up in our institution. The OS of these 79 patients was >1 month. Thereafter, a TMA was constructed for the 79 samples using 1.5 mm tissue cores. All tissues were approved by the Clinical Research Ethics Committee of the First Affiliated Hospital of Nanjing Medical University. Informed consent was obtained from all participants.

### Statistical Analysis

Independent experiments were performed three times in each figure. The comparison between two groups was conducted by independent Student’s t-test. SPSS 21.0 software were used to perform all the analyses. All data were expressed as mean ± SD. Differences were considered statistically significant at P < 0.05.

## Results

### CD8+ T Cells Predicts Poor Prognosis in PDAC

**Figure 1:**
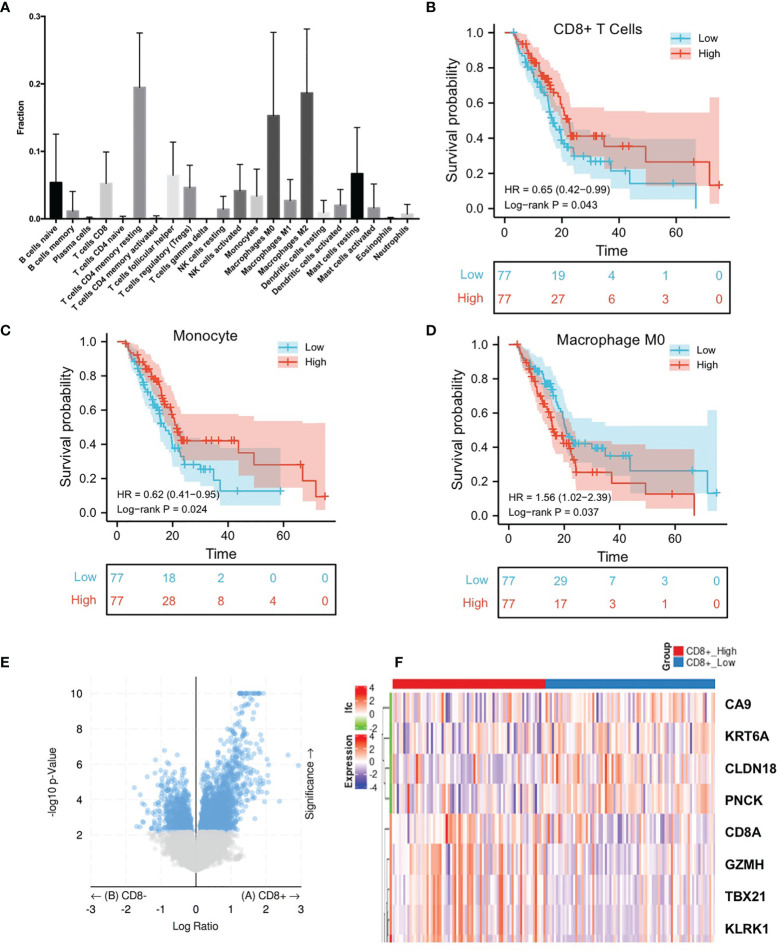
Comprehensive analysis of TME components of TCGA pancreatic cancer patients indicate the prognostic value of CD8+ T cells. **(A)** TCGA pancreatic cancer transcriptome sequencing data CIBERSORT analysis results show the main infiltrating immune cells in pancreatic cancer. **(B–D)** TCGA data suggest that the prognosis of patients with pancreatic cancer with high CD8+ T cell infiltration, high monocyte infiltration, and low macrophage M0 is significantly better. **(E)** Volcano plots of DEGs between CD8+ T cells high infiltration group and low group. X-axis indicates the fold change (log scaled), whereas the Y-axis shows the p values (log scaled). Each symbol represents a different gene, and the red/blue color of the symbols categorize the upregulated/downregulated genes falling under different criteria (p value and fold change threshold). p value <0.05 is considered as statistically significant, whereas fold change >1 is set as the threshold. **(F)** Heatmaps of the top 8 DEGs between between CD8+ T cells high infiltration group and low group.

We downloaded the transcriptome and clinical data of TCGA pancreatic cancer cohort. Based on CIBERSORT, we found that the major TME components for PDAC were resting memory CD4+ T cells, CD8+ T cells, follicular helper T cells, M2 Macrophages, M0 Macrophages and resting mast cells. Low infiltration of plasma cells, naive CD4+ T cells, activated CD4+ memory T cells, eosinophils and neutrophils was observed for PDAC patients (**Figure 1A**). According to the bioinformatics analysis of TCGA transcriptome data, among the 22 immune microenvironment components, low CD8+ T cell infiltration is significantly correlated with poor prognosis of pancreatic cancer (**Figure 1B**). We also found that monocyte and macrophage m0 were associated with patients outcome (**Figures 1C, D**). No other components were with significant effect on the overall survival (**Figure S1**).

### CA9 Expression Was Associated With CD8+ T Cells Infiltration

**Figure 2:**
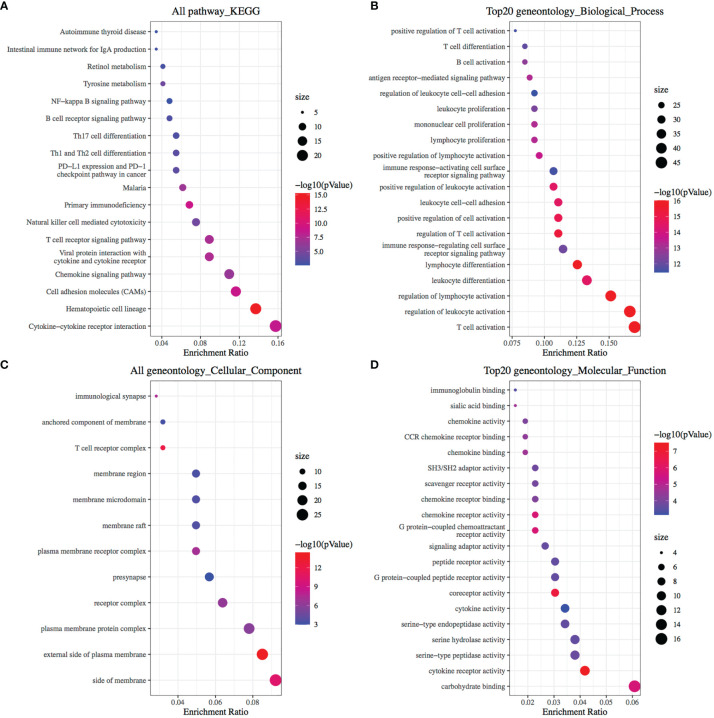
Enrichment Analysis of DEGs identified from comparing CD8+ T cells high infiltration group and low infiltration group. **(A)** Bubble plot of significant KEGG pathways. X-axis indicates the gene ratio, whereas the Y-axis shows the most enriched KEGG pathways. And the size of bubble presents genes number, and the color of bubble presents the P value. **(B–D)** GO analyses of the DEGs according to their biological process, cellular component and molecular function. X-axis indicates the gene number, whereas the Y-axis shows the most enriched GO terms.

After analyzing the differences in the gene expression of pancreatic cancer tissues between the CD8+ T cell high and low infiltration groups, there were 308 significant DEGs (|log2 FoldChange|>1.0, p <0.05), of which 268 genes were up-regulated in CD8+ T cell high infiltration group and 40 genes were down-regulated (**Figures 1E, F** and **Supplementary Table S1**). The KEGG analysis demonstrated that there were 19 significant pathways (FDR<0.05) (**Figure 2A**), including Autoimmune thyroid disease, Intestinal immune network for IgA production, Retinol metabolism, etc (**Figure 2B**). There were 12 GO Cellular Components including immunological synapse anchored component of membrane, T cell receptor complex, etc (**Figure 2C**). GO Molecular Functions were also enriched (**Figure 2D**).

**Figure 3:**
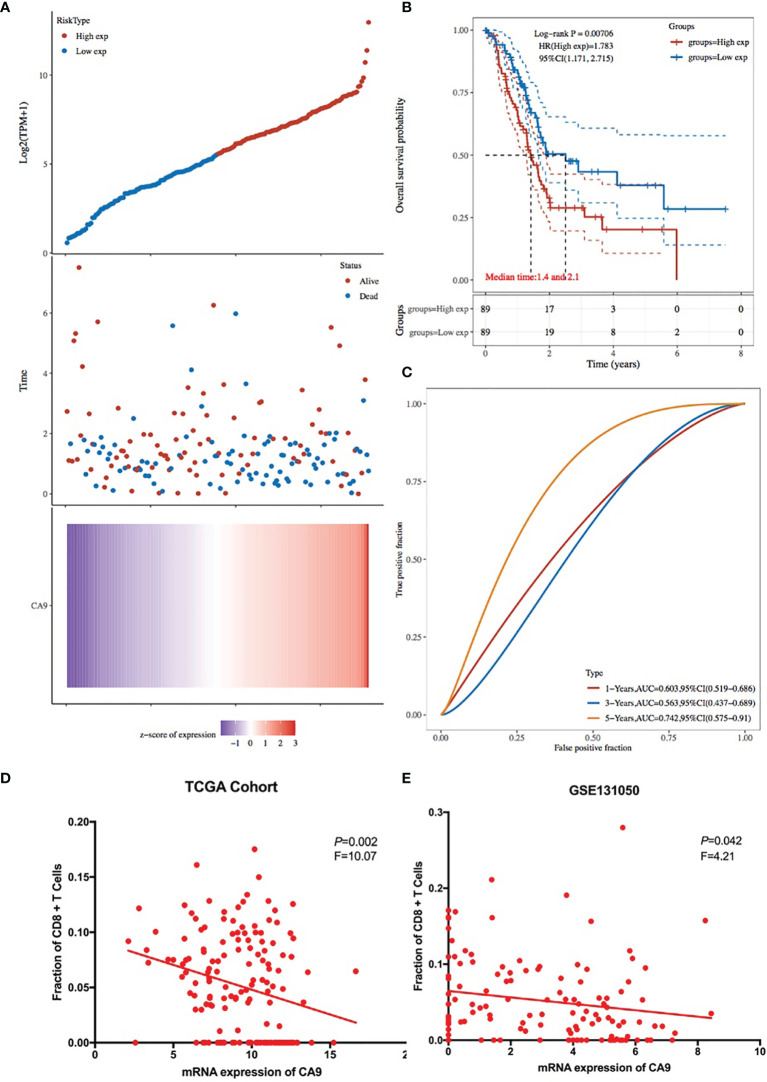
Prognostic value of CA9 and its association with CD8+ T cells infiltration from TCGA and GEO pancreatic cancer dataset. **(A)** Risk score plot and heatmap of CA9 among TCGA pancreatic cancer patients. **(B)** Survival time and status for patients with high and low expression of CA9. **(C)** ROC analysis of overall survival for the CA9 gene signature in TCGA cohort. **(D, E)**. TCGA cohort data suggest that CD8+ T cell infiltration and CA9 expression are closely related, and GEO dataset GSE131050 confirms this phenomenon.

**Figure 4:**
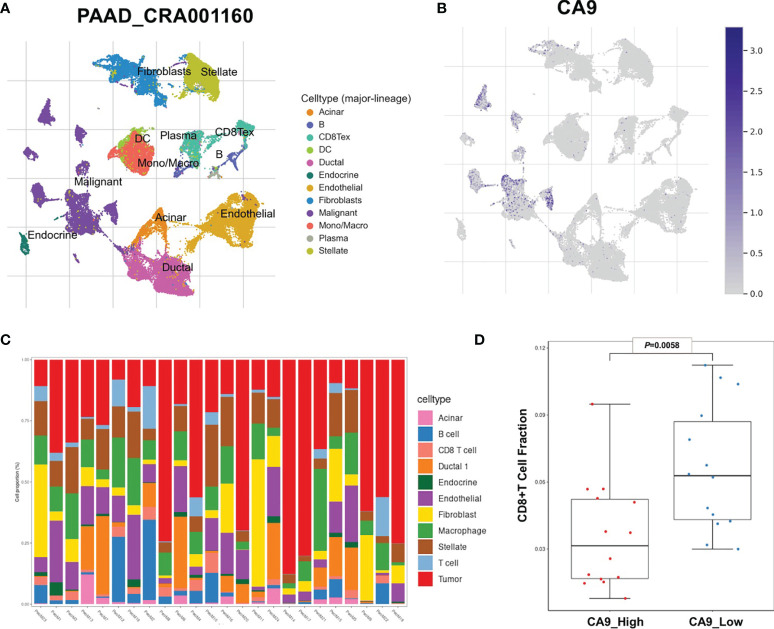
Single-cell sequencing data suggests that CA9 is mainly expressed in cancer cells and is related to the proportion of CD8+ T cells. **(A, B)** Pancreatic cancer single-cell sequencing dataset PAAD_CRA001160 analysis results show that CA9 is mainly expressed in pancreatic cancer cells. **(C)** Analysis of cell subsets in PAAD_CRA001160 single-cell sequencing dataset. **(D)** CA9 expression of the cancer cell cluster in the single-cell dataset is correlated with CD8+ T cell infiltration.

It was found that CA9 expression was most significantly negatively correlated with the proportion of CD8+ T cell infiltration (**Figures 1D**, **3D**). The GEO data set verified this phenomenon (**Figure 3E**). To further validate the relationship between cancer cells CA9 expression and CD8+ T cells infiltration, scRNA-seq dataset CRA001160 was reanalyzed. It was shown that CA9 is mainly expressed in pancreatic cancer cell cluster (**Figures 4A, B**). CA9 expression of cancer cell subgroup is related to CD8+ T cell infiltration (**Figures 4C, D**).

### CA9 Expression Was Associated With Outcome of Pancreatic Cancer Patients

KM survival analysis according to the TCGA PDAC dataset demonstrated that patients with lower levels of CA9 expression had a superior OS than those with higher levels of CA9 expression (P < 0.01) (**Figure 3B**). The risk score for each sample was then calculated based on the expression levels of CA9 (**Figure 3A**). Time-dependent receiver operating characteristics (ROC) analysis was performed. The area under the ROC curve (AUC) of the risk score of 1-year, 3-year, and 5- year survival was 0.603, 0.563, and 0.742 (**Figure 3C**). suggesting the CA9 prognostic signature for PDAC was reliable. Moreover, we further proved that CA9 overexpression positively correlated with lymphatic metastasis (**Table 1**) and a worse OS in PDAC patients from TMA data (**Figures 6B, C**).

**Table 1:** Clinical characteristics of patients included in TMA analysis.

Characteristics	CA9_Low (N = 39)	CA9_High (N = 40)	*p* value
Gender			1.00
F	16(20.25%)	16(20.25%)	
M	23(29.11%)	24(30.38%)	
Age			0.75
Mean ± SD	61.69 ± 9.80	60.88 ± 12.32	
Median[min-max]	61.00[41.00,77.00]	63.50[32.00,80.00]	
T stage			0.59
1	3(3.80%)	1(1.27%)	
2	5(6.33%)	3(3.80%)	
3	28(35.44%)	33(41.77%)	
4	3(3.80%)	3(3.80%)	
N stage			**0.03**
0	28(35.44%)	18(22.78%)	
1	11(13.92%)	22(27.85%)	
M stage			0.99
0	38(48.10%)	40(50.63%)	
1	1(1.27%)	0(0.0e+0%)	
Perineural invasion			0.69
no	12(15.19%)	15(18.99%)	
yes	27(34.18%)	25(31.65%)	
Lymphovascular invasion			0.33
no	36(45.57%)	33(41.77%)	
yes	3(3.80%)	7(8.86%)	

Bold value means statistically significant.

### CA9 Induces Acidic Microenvironment and Inhibits CD8+ T Cells

**Figure 5:**
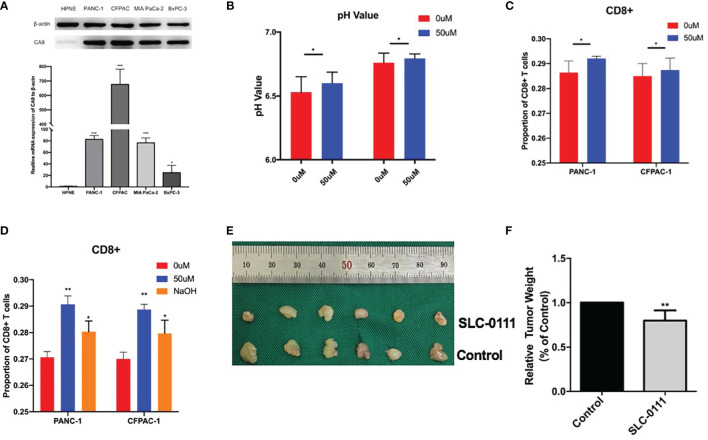
CA9 induce acidic microenvironment and inhibit CD8+ T cells. **(A)** The expression of CA9 in pancreatic cancer cell lines is significantly higher than that of normal pancreatic duct cell lines. **(B)** After adding CA9 specific inhibitor SLC-0111 (50uM), the pH value of the pancreatic cancer culture supernatant is significantly increased. **(C)** Adding CA9 inhibitor to pancreatic cancer cell line and PBMC cell co-culture can up-regulate CD8+ T cells. Using NaOH to adjust the pH value to the same as the CA9 inhibitor group partially up-regulate CD8+ T cells. **(D, E)** *In vivo* experiments indicate that the C57BL/6 mouse model of tumor formation was randomly divided into two groups after tumor formation, and intraperitoneal injection of normal saline and SLC-0111 for three weeks respectively. Tumors of mice injected with SLC-0111 were significantly smaller than the control group. * means p<0.05 and ** means p<0.01.

**Figure 6:**
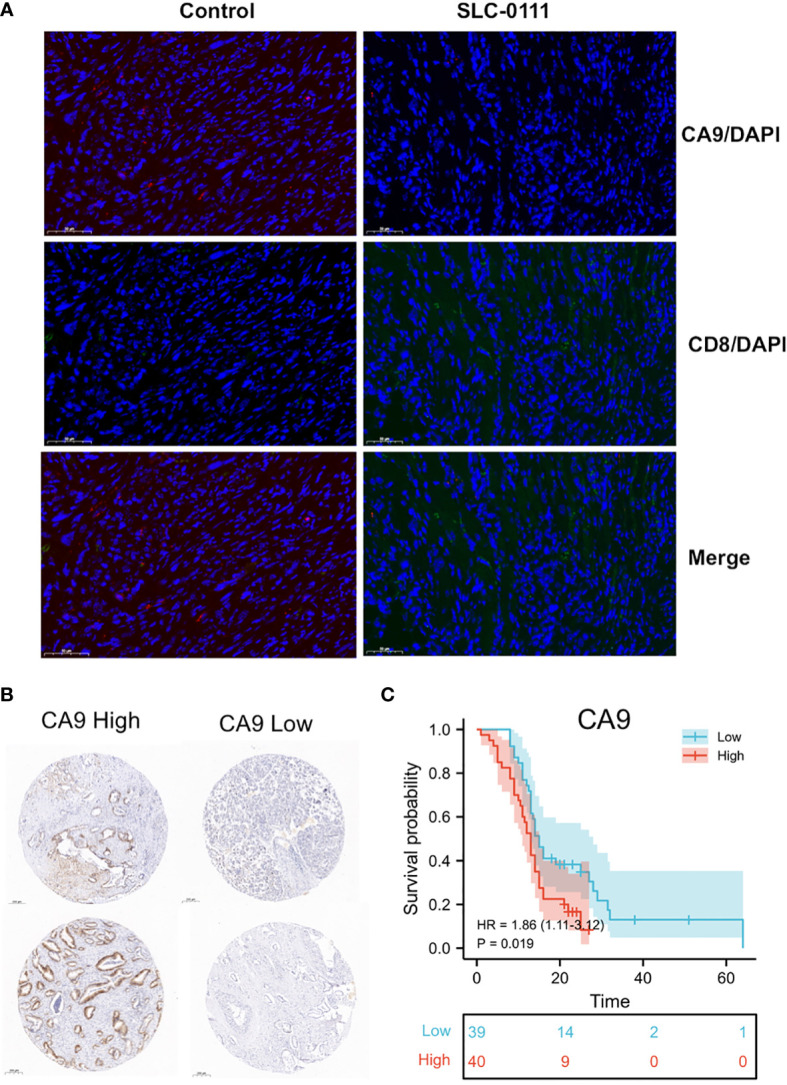
**(A)** SLC-0111 inhibits CA9 expression and up-regulate CD8+ T cells *in vivo*. **(B)** TMA IHC analysis showed that CA9 high expression is associated with worse survival (Examples of CA9 expression in pancreatic cancer patients). **(C)** CA9 expression is significantly associated with overall survival of patients.

We further evaluated the effect of CA9 on CD8+ T cells in pancreatic cancer at the cellular level. The results showed that CA9 is widely expressed in pancreatic cancer cell lines, such as PANC-1, CFPAC-1, MIA Paca-2, etc (**Figure 5A**). CA9 inhibitor SLC-0111 increased the pH value in the pancreatic cancer cell culture supernatant (**Figure 5B**). The culture supernatant of each group was co-cultured with PBMC from healthy volunteers and the proportion of CD8+ T cells is higher than that of the control group (**Figure 5C**). After adjusting to the same pH value with NaOH, the proportion and function of CD8+ T cells can also be partially increased (**Figure 5C**). *In vivo* experiments indicate that the tumors of mice injected with SLC-0111 were significantly smaller than the control group (**Figures 5D, E**). Multi-color immunofluorescence to investigate the effect of SLC-0111 on CA9 expression and CD8+ T cells infiltration in mouse model showed that SLC-0111 inhibits CA9 expression and up-regulate CD8+ T cells *in vivo* (**Figure 6A**).

### CA9 Expression Was Associated With Immune Checkpoints, Tumor Mutation Burden (TMB) and Ferroptosis-Related mRNA

**Figure 7:**
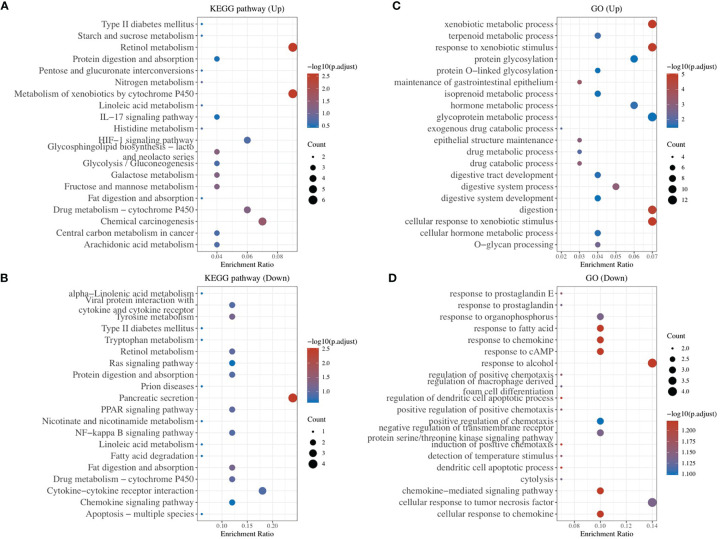
Enrichment Analysis of DEGs identified from comparing CA9 high expression group and low expression group. **(A, B)** Bubble plot of significant KEGG pathways. X-axis indicates the gene ratio, whereas the Y-axis shows the most enriched KEGG pathways. And the size of bubble presents genes number, and the color of bubble presents the P value. **(C, D)** GO analyses of the DEGs according to their biological process, cellular component and molecular function. X-axis indicates the gene number, whereas the Y-axis shows the most enriched GO terms.

**Figure 8:**
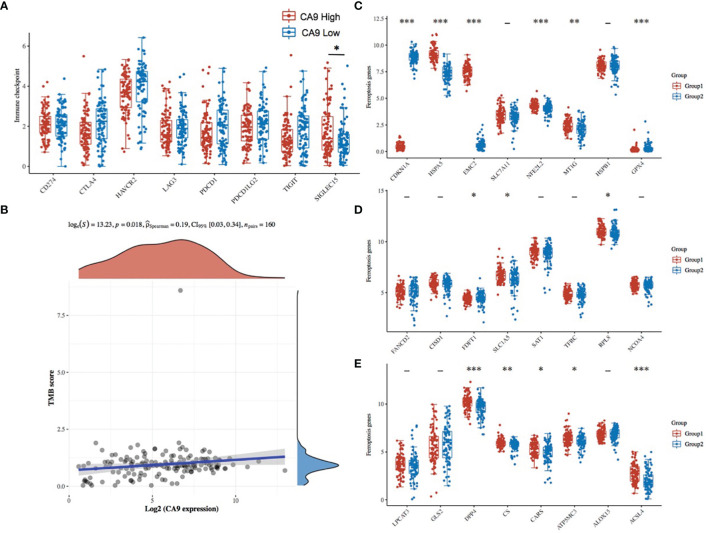
The association between CA9 expression and immune checkpoints, tumor mutation burden and ferroptosis-related genes from TCGA dataset. **(A)** Comparison of immune checkpoints expression among CA9-high tumor tissues and CA9-low tumor tissues. **(B)** Correlation analysis of CA9 expression and TMB. The horizontal axis in the figure represents the expression distribution of the gene, and the ordinate is the expression distribution of the TMB score. The density curve on the right represents the distribution trend of the TMB score; the upper density curve represents the distribution trend of the gene; the top side The value represents the correlation p value, correlation coefficient and correlation calculation method. **(C-E)**. The expression distribution of Ferroptosis-related mRNA in CA9-high and CA9-low tumor tissues, where the horizontal axis represents different mRNA, the vertical axis represents the mRNA expression distribution, where different colors represent different groups, and the upper left corner represents the significance p-value test method.Asterisks represent levels of significance *p < 0.05, **p < 0.01, ***p < 0.001.

To explore other mechanisms of CA9 regulating CD8+ T cells, DEGs analysis and comprehensive analysis of immune checkpoints, tumor mutation burden (TMB), Ferroptosis-related mRNA and m6A related mRNAs were performed (**Table S2**, **Figures 7**, **8**, **S3**). The KEGG analysis demonstrated that immune-related pathway including IL−17 signaling pathway, NF−kappa B signaling pathway, cytokine and cytokine receptor Tyrosine metabolism, etc (**Figures 7A, B**). GO terms were also enriched (**Figures 7C, D**). The association analysis between CA9 expression and immune checkpoints showed that SIGLEC15 was significantly higher in CA9 high group (**Figure 8A**). CA9 was positively correlated with TMB score in TCGA PDAC patients (**Figure 8B**). Significant differentially expressed ferroptosis-related genes included CDKN1A, HSPA5, EMC2, NFE2L2, GPX4, DPP4, etc (**Figure 8C**).

## Discussion

The immune microenvironment of pancreatic cancer has the characteristics of strong heterogeneity, and the degree of infiltration and functional status of each T cell subgroup is different (8, 9), and exploring the underlying mechanism of this phenomenon is of great significance for the treatment of pancreatic cancer. Our previous study found that the location of the tumor may affect the infiltration of immune cells (10). Previous studies showed that the immunosuppressive microenvironment composed of fibroblasts and proliferative matrix would limit T cell infiltration (11). In addition, there are various soluble immunosuppressive molecules (such as TGF-β, IL-10, IL-23, IDO and VEGF-A, etc.) and immunosuppressive cells (Tregs, MDSCs and TAMs) in the tumor microenvironment of pancreatic cancer. Etc.) can cause immune effector cells to be in an imbalanced state, forming a unique immunosuppressive environment for pancreatic cancer (12). In addition, studies have found that the acidic microenvironment of tumors can inhibit the function of immune cells, while neutralizing the acidic microenvironment can inhibit the growth or metastasis of a variety of tumors (13), which may be related to the infiltration of immune active CD8+ T cells in the tumor.

To date, members of the carbonic anhydrase (CA) family that have been identified in human pancreas include CA1, CA2, CA4, CA5B, CA6, CA9, and CA12. CA9 catalyzes a simple physiological reaction, that is, carbon dioxide reversibly hydrates to bicarbonate ions and protons. Therefore, CA9 embedded in the cell membrane is an important part of the tumor pH regulation mechanism (14). The expression pattern of CA9 is significantly different from other CA family members: CA9 can only be found in a few normal tissues, but its abnormal expression is closely related to many cancer types (15). Compared with normal tissues and precancerous lesions, CA9 expression is higher in pancreatic cancer tissues, and it is positively correlated with tumor size and stage (16). Recent evidence shows that the enzyme activity of CA9 and the related acidic microenvironment not only play a key role in the survival of cancer cells under hypoxia and their migration and invasion characteristics, but also play a key role in immune surveillance (17). The highly acidic immune microenvironment can weaken anti-tumor immunity through a variety of mechanisms, such as inhibiting the infiltration and cytolytic activity of CD8+ T cells, inhibiting the production of cytokines, and promoting the immunosuppressive phenotype of macrophages (18).

Our study showed that CA9-related acidic microenvironment can directly affect the function of CD8+ T cells, and when the pH factor is controlled, the function of CD8+ T cells is only partially restored, indicating that CA9 may promote CD8+ T cell disability through other mechanisms. Our study found that immune checkpoint SIGLEC15 (Sialic Acid Binding Ig Like Lectin 15) may be involved in the inhibition of CD8+ T cells by the CA9-related microenvironment.

In recent years, immune checkpoint therapy represented by PD-1/PD-L1 inhibitors has not been effective in clinical trials for the treatment of pancreatic cancer (4). Even targeting multiple T cell immune checkpoint proteins, such as PD-L1 and CTLA-4, failed to significantly improve the patient’s response (19). Under the situation of poor immunotherapy for pancreatic cancer, in 2019 Nature Medicine published a new immunosuppressive molecule SIGLEC15 (20). SIGLEC15 is widely expressed in a variety of tumor cells. Studies have shown that SIGLEC15 expressed on the surface of tumor cells can bind to the corresponding receptors on the surface of CD8+ T cells, thereby inhibiting its function (21). Studies have found that the expression of PD-L1 and SIGLEC15 are mutually exclusive, indicating that SIGLEC15 targeted therapy has certain application value in patients who do not respond to anti-PD-1/PD-L1 treatment (21). Further study is needed to explore the potential mechanism of CA9 regulating CD8+ T cells.

## Conclusion

Pancreatic cancer cells may inhibit the infiltration of CD8+ T cells through CA9. Further exploration of its related mechanisms can be used to explore the immune escape pathway of pancreatic cancer and provides new perspectives immune targeted therapy.

## Data Availability Statement

The datasets presented in this study can be found in online repositories. The names of the repository/repositories and accession number(s) can be found in the article/**Supplementary Material**.

## Ethics Statement

The studies involving human participants were reviewed and approved by Clinical Research Ethics Committee of The First Affiliated Hospital of Nanjing Medical University. The patients/participants provided their written informed consent to participate in this study. The animal study was reviewed and approved by Animal ethics committee of Nanjing Medical University.

## Author Contributions

QL, YP, and LY participated in the conceptualization and design. YL and CC interpreted the reported experiments or results. All authors participated in the acquisition and analysis of data. LY, YP, and YL participated in drafting and revising the manuscript. All the authors revised the manuscript and agreed with the manuscript’s results and conclusion. KJ and YM supervised the study. All authors contributed to the article and approved the submitted version.

## Funding

This work was supported by the grant from the National Science Foundation for Young Scientists of China (Grant No. 81802408), the Innovation Capability Development Project of Jiangsu Province (No. BM2015004), Jiangsu Province “333” Project (2019-RS19), Jiangsu Biobank of Clinical Resources (BM2015004) and Young Scholars Fostering Fund of the First Affiliated Hospital of Nanjing Medical University (to LY, PY2021042).

## Conflict of Interest

The authors declare that the research was conducted in the absence of any commercial or financial relationships that could be construed as a potential conflict of interest.

## Publisher’s Note

All claims expressed in this article are solely those of the authors and do not necessarily represent those of their affiliated organizations, or those of the publisher, the editors and the reviewers. Any product that may be evaluated in this article, or claim that may be made by its manufacturer, is not guaranteed or endorsed by the publisher.

## Supplementary Material

Supplementary Figure 1 Prognostic value of other immune microenvironment components in TCGA PDAC patients.

Supplementary Figure 2 Volcano plots of DEGs between CA9 high expression group and low expression group.

Supplementary Figure 3 The expression distribution of m6A related mRNA in CA9-high tumor tissues (group 1), CA9-low tumor tissues (group 2) and normal tissues, where the horizontal axis represents different mRNA, the vertical axis represents the mRNA expression distribution, where different colors represent different groups, and the upper left corner represents the significance p-value test method. Asterisks represent levels of significance *p < 0.05, **p < 0.01, ***p < 0.001.

## References

[1] Cao Y Li L Xu M Feng Z Sun X Lu J. The ChinaMAP Analytics of Deep Whole Genome Sequences in 10,588 Individuals. Cell Res (2020) 30(9):717–31. doi: 10.1038/s41422-020-0322-9 PMC7609296 32355288

[2] Strobel O Neoptolemos J Jäger D Büchler MW. Optimizing the Outcomes of Pancreatic Cancer Surgery. Nat Rev Clin Oncol (2019) 16(1):11–26. doi: 10.1038/s41571-018-0112-1 30341417

[3] Michl P Gress TM. Current Concepts and Novel Targets in Advanced Pancreatic Cancer. Gut (2013) 62(2):317–26. doi: 10.1136/gutjnl-2012-303588 23112132

[4] Leinwand J Miller G. Regulation and Modulation of Antitumor Immunity in Pancreatic Cancer. Nat Immunol (2020) 21(10):1152–9. doi: 10.1038/s41590-020-0761-y 32807942

[5] Kirby MK Ramaker RC Gertz J Davis NS Johnston BE Oliver PG. RNA Sequencing of Pancreatic Adenocarcinoma Tumors Yields Novel Expression Patterns Associated With Long-Term Survival and Reveals a Role for ANGPTL4. Mol Oncol (2016) 10(8):1169–82. doi: 10.1016/j.molonc.2016.05.004 PMC5423196 27282075

[6] Peng J Sun BF Chen CY Zhou JY Chen YS Chen H. Single-Cell RNA-Seq Highlights Intra-Tumoral Heterogeneity and Malignant Progression in Pancreatic Ductal Adenocarcinoma. Cell Res (2019) 29(9):725–38. doi: 10.1038/s41422-019-0195-y PMC6796938 31273297

[7] Peng YP Zhu Y Zhang JJ Xu ZK Qian ZY Dai CC. Comprehensive Analysis of the Percentage of Surface Receptors and Cytotoxic Granules Positive Natural Killer Cells in Patients With Pancreatic Cancer, Gastric Cancer, and Colorectal Cancer. J Trans Med (2013) 11:262. doi: 10.1186/1479-5876-11-262 PMC3854023 24138752

[8] Stromnes IM Hulbert A Pierce RH Greenberg PD Hingorani SR. T-Cell Localization, Activation, and Clonal Expansion in Human Pancreatic Ductal Adenocarcinoma. Cancer Immunol Res (2017) 5(11):978–91. doi: 10.1158/2326-6066.cir-16-0322 PMC5802342 29066497

[9] Li J Byrne KT Yan F Yamazoe T Chen Z Baslan T. Tumor Cell-Intrinsic Factors Underlie Heterogeneity of Immune Cell Infiltration and Response to Immunotherapy. Immun (2018) 49(1):178–93. doi: 10.1016/j.immuni.2018.06.006 PMC6707727 29958801

[10] Yin L Xiao L Gao Y Wang G Gao H Peng Y. Comparative Bioinformatical Analysis of Pancreatic Head Cancer and Pancreatic Body/Tail Cancer. Med Oncol (Northwood London England) (2020) 37(5):46. doi: 10.1007/s12032-020-01370-0 32277286

[11] Ene-Obong A Clear AJ Watt J Wang J Fatah R Riches JC. Activated Pancreatic Stellate Cells Sequester CD8+ T Cells to Reduce Their Infiltration of the Juxtatumoral Compartment of Pancreatic Ductal Adenocarcinoma. Gastroenterol (2013) 145(5):1121–32. doi: 10.1053/j.gastro.2013.07.025 PMC3896919 23891972

[12] Balli D Rech AJ Stanger BZ Vonderheide RH. Immune Cytolytic Activity Stratifies Molecular Subsets of Human Pancreatic Cancer. Clin Cancer Res an Off J Am Assoc Cancer Res (2017) 23(12):3129–38. doi: 10.1158/1078-0432.ccr-16-2128 PMC12164831 28007776

[13] Hashim AAI Abrahams D Xu L Centeno B Sunassee E Abddelgader R. Abstract 5932: Targeting Tumor Acidity With the LDHA Inhibitor (FX11) and CAIX Inhibitor (DH348) Overcomes Resistance to PD-1 Blockade and Inhibits Metastasis in a Pancreatic Cancer Model. Cancer Res (2017) 77(13 Supplement):5932–2. doi: 10.1158/1538-7445.am2017-5932

[14] Svastova E Witarski W Csaderova L Kosik I Skvarkova L Hulikova A. Carbonic Anhydrase IX Interacts With Bicarbonate Transporters in Lamellipodia and Increases Cell Migration *via* Its Catalytic Domain. J Biol Chem (2012) 287(5):3392–402. doi: 10.1074/jbc.M111.286062 PMC3270993 22170054

[15] Baniak N Flood TA Buchanan M Dal Cin P Hirsch MS Carbonic anhydrase IX. (CA9) Expression in Multiple Renal Epithelial Tumour Subtypes. Histopathol (2020) 77(4):659–66. doi: 10.1111/his.14204 32639054

[16] Li Y Dong M Sheng W Huang L. Roles of Carbonic Anhydrase IX in Development of Pancreatic Cancer. Pathol Oncol Res POR (2016) 22(2):277–86. doi: 10.1007/s12253-015-9935-6 26224207

[17] McDonald PC Chafe SC Dedhar S. Overcoming Hypoxia-Mediated Tumor Progression: Combinatorial Approaches Targeting pH Regulation, Angiogenesis and Immune Dysfunction. Front Cell Dev Biol (2016) 4:27. doi: 10.3389/fcell.2016.00027 27066484 PMC4814851

[18] Pilon-Thomas S Kodumudi KN El-Kenawi AE Russell S Weber AM Luddy K. Neutralization of Tumor Acidity Improves Antitumor Responses to Immunotherapy. Cancer Res (2016) 76(6):1381–90. doi: 10.1158/0008-5472.can-15-1743 PMC4829106 26719539

[19] O'Reilly EM Oh DY Dhani N Renouf DJ Lee MA Sun W. Durvalumab With or Without Tremelimumab for Patients With Metastatic Pancreatic Ductal Adenocarcinoma: A Phase 2 Randomized Clinical Trial. JAMA Oncol (2019) 5(10):1431–8. doi: 10.1001/jamaoncol.2019.1588 PMC6647002 31318392

[20] Wang J Sun J Liu LN Flies DB Nie X Toki M. Siglec-15 as an Immune Suppressor and Potential Target for Normalization Cancer Immunotherapy. Nat Med (2019) 25(4):656–66. doi: 10.1038/s41591-019-0374-x PMC7175920 30833750

[21] Sun J Lu Q Sanmanmed MF Wang J. Siglec-15 as an Emerging Target for Next-Generation Cancer Immunotherapy. Clin Cancer Res an Off J Am Assoc Cancer Res (2021) 27(3):680–8. doi: 10.1158/1078-0432.ccr-19-2925 PMC9942711 32958700

