# Supplementary figures and images for: CA9-Related Acidic Microenvironment Mediates CD8+ T Cell Related Immunosuppression in Pancreatic Cancer
Source: Front Oncol. 2022 Jan 27;11:832315. doi: 10.3389/fonc.2021.832315 (PMC8828571; doi:10.3389/fonc.2021.832315)

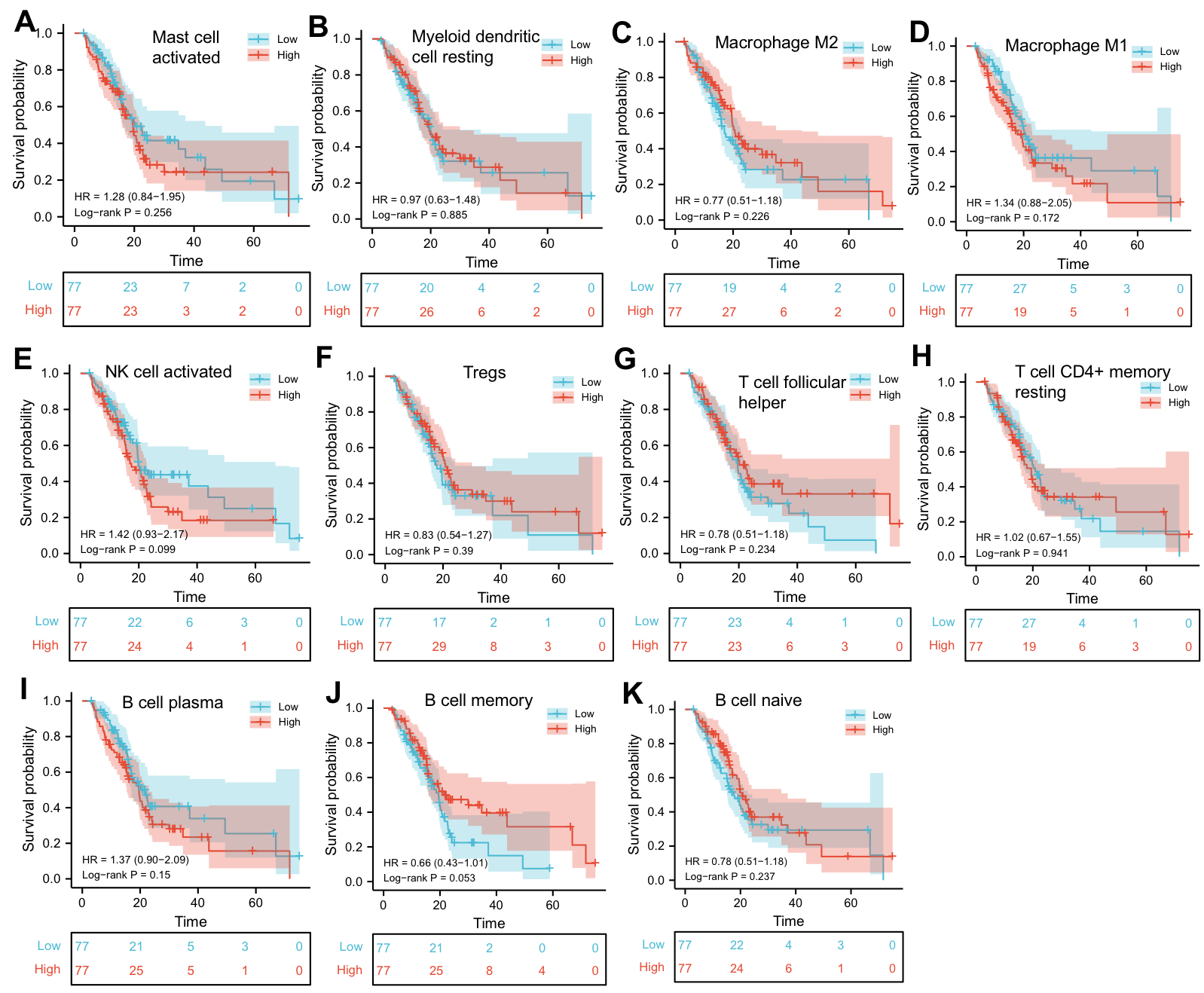

Supplement: Supplementary Figure 1 — Prognostic value of other immune microenvironment components in TCGA PDAC patients. [file Image_1.png]

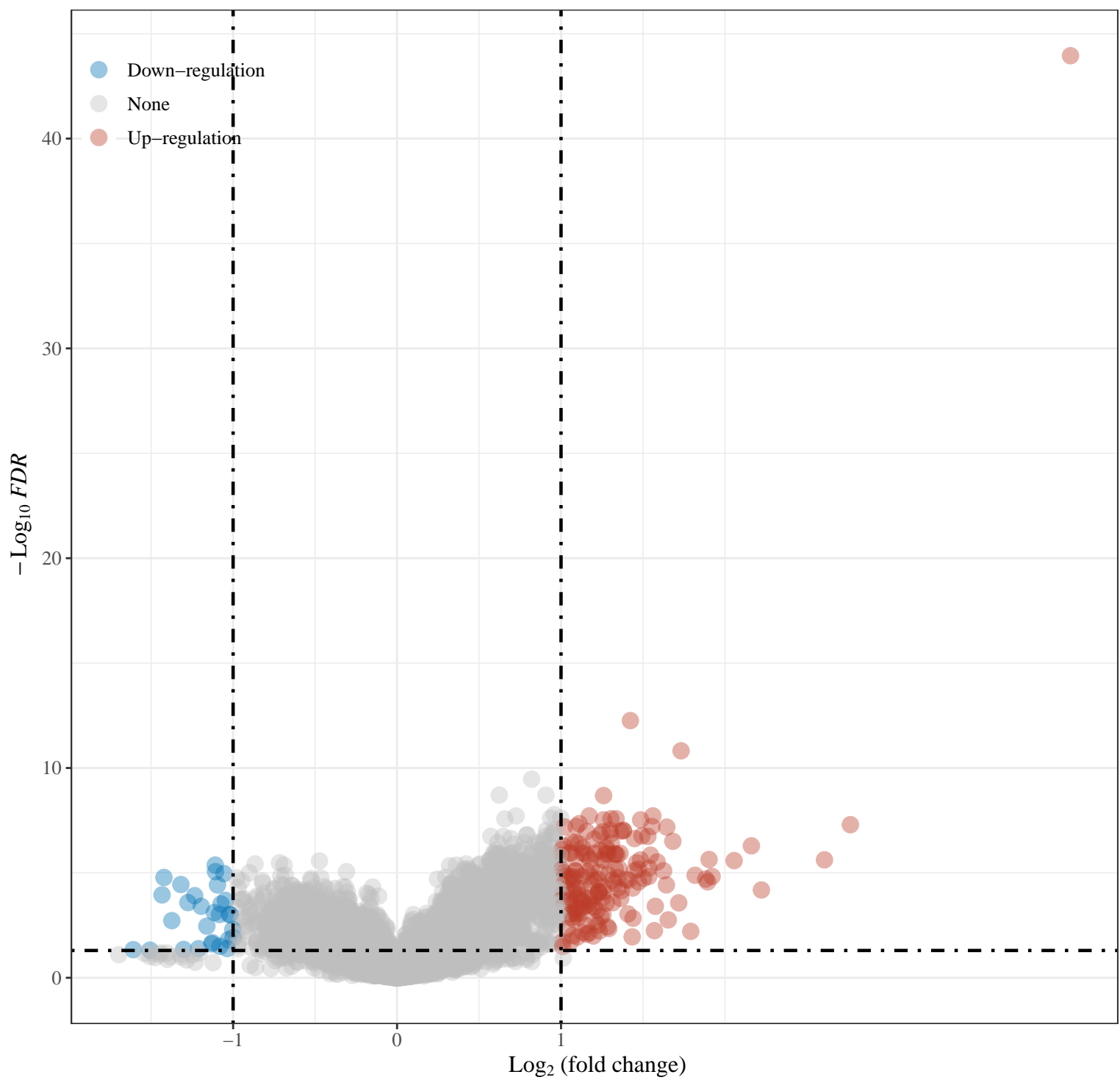

Supplement: Supplementary Figure 2 — Volcano plots of DEGs between CA9 high expression group and low expression group. [file Image_2.pdf]

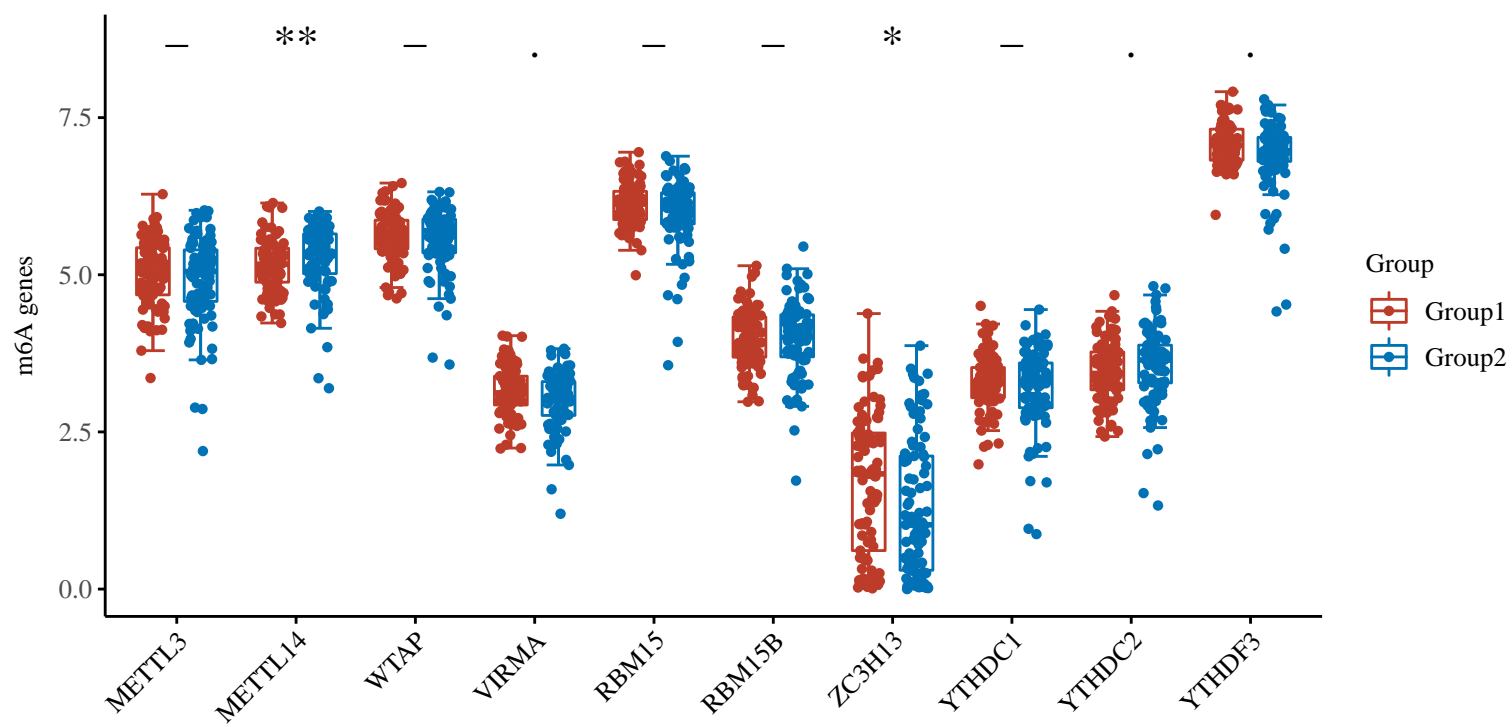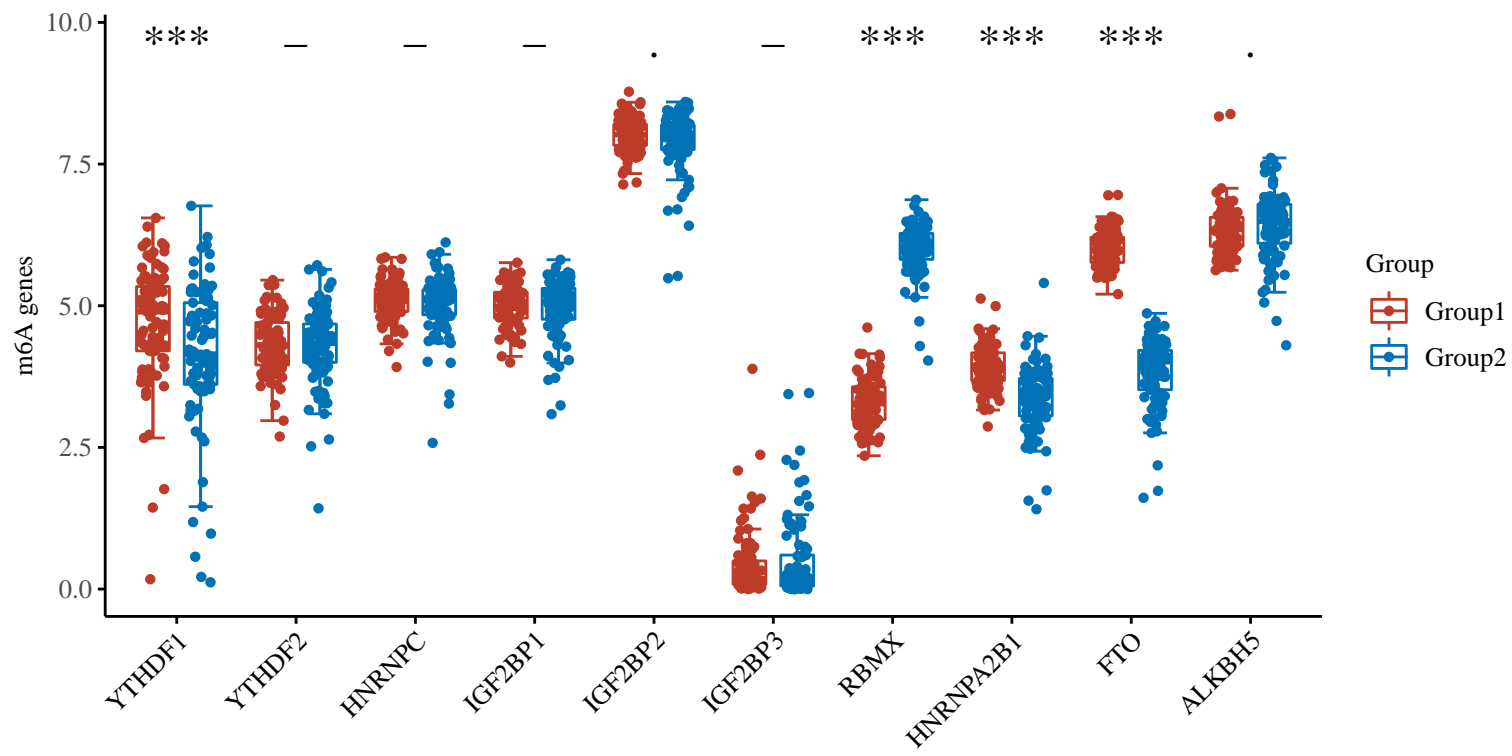

Supplement: Supplementary Figure 3 — The expression distribution of m6A related mRNA in CA9-high tumor tissues (group 1), CA9-low tumor tissues (group 2) and normal tissues, where the horizontal axis represents different mRNA, the vertical axis represents the mRNA expression distribution, where different colors represent different groups, and the upper left corner represents the significance p-value test method. Asterisks represent levels of significance *p < 0.05, **p < 0.01, ***p < 0.001. [file Image_3.pdf]
